# Insights into the Regulatory Characteristics of the Mycobacterial Dephosphocoenzyme A Kinase: Implications for the Universal CoA Biosynthesis Pathway

**DOI:** 10.1371/journal.pone.0021390

**Published:** 2011-06-24

**Authors:** Guneet Walia, Avadhesha Surolia

**Affiliations:** 1 Molecular Biophysics Unit, Indian Institute of Science, Bangalore, India; 2 National Institute of Immunology, New Delhi, India; Queen Mary University of London, United Kingdom

## Abstract

Being vastly different from the human counterpart, we suggest that the last enzyme of the *Mycobacterium tuberculosis* Coenzyme A biosynthetic pathway, dephosphocoenzyme A kinase (CoaE) could be a good anti-tubercular target. Here we describe detailed investigations into the regulatory features of the enzyme, affected via two mechanisms. Enzymatic activity is regulated by CTP which strongly binds the enzyme at a site overlapping that of the leading substrate, dephosphocoenzyme A (DCoA), thereby obscuring the binding site and limiting catalysis. The organism has evolved a second layer of regulation by employing a dynamic equilibrium between the trimeric and monomeric forms of CoaE as a means of regulating the effective concentration of active enzyme. We show that the monomer is the active form of the enzyme and the interplay between the regulator, CTP and the substrate, DCoA, affects enzymatic activity. Detailed kinetic data have been corroborated by size exclusion chromatography, dynamic light scattering, glutaraldehyde crosslinking, limited proteolysis and fluorescence investigations on the enzyme all of which corroborate the effects of the ligands on the enzyme oligomeric status and activity. Cysteine mutagenesis and the effects of reducing agents on mycobacterial CoaE oligomerization further validate that the latter is not cysteine-mediated or reduction-sensitive. These studies thus shed light on the novel regulatory features employed to regulate metabolite flow through the last step of a critical biosynthetic pathway by keeping the latter catalytically dormant till the need arises, the transition to the active form affected by a delicate crosstalk between an essential cellular metabolite (CTP) and the precursor to the pathway end-product (DCoA).

## Introduction

More than a century, a vaccine and several chemotherapeutic agents later, *Mycobacterium tuberculosis* continues its deadly march claiming several thousand lives each year [1], [2]. With mycobacteria investing a major part of their coding capacity towards fatty acid synthesis, there being a whopping 250 distinct enzymes involved in fatty acid metabolism and 9% of all cellular activities employing Coenzyme A (CoA) as a cofactor (BRENDA database), targeting the tubercular CoA biosynthesis holds potential in globally impairing the pathogen, as CoA is involved in a variety of critical cellular processes, the chief being the synthesis of the cell wall mycolic acids [3]. This ubiquitous, indispensable cofactor, along with its precursor, 4′-phosphopantetheine (prosthetic group incorporated by carrier proteins), functions as an acyl group carrier and carbonyl-activating group for Claisen reactions as well as for amide-, ester-and thioester-forming reactions in the cell [4]. CoA and its thioesters participate directly in the biosynthesis of five amino acids and indirectly in macromolecular biosynthesis through glutamate synthesis via the tricarboxylic acid cycle [5]. CoA is an important metabolite for cellular growth demonstrated by the fact that a fall in its concentration below 5 pmol/10^8^ cells leads to growth stasis [6]. The choice of the CoA biosynthetic pathway for investigation is lent credence by the fact that this pathway is essential in a majority of the prokaryotic pathogens, any deletions leading to growth stasis or lethality. The fact that these organisms are unable to take up either CoA or the phosphorylated reaction intermediates exogenously further corroborates the choice of this pathway as a potential drug target. Interestingly, evolutionary evidence points out that this universal biosynthetic pathway branched out early during the evolutionary history of life on earth and the present day eukaryotic and prokaryotic CoA-synthetic machinery differs vastly, with major differences in the basic architecture of the pathway and the regulation of individual steps [7].

Considering the plethora of critical roles this cofactor plays, regulation of its biosynthetic pathway has been under scrutiny for a long time. Even before the mechanistic details of the first enzyme of the CoA pathway were known, it was demonstrated that this step forms an important regulatory site of the biosynthetic pathway being potently feedback regulated by the end-product of the pathway and to a lesser extent, by its thioesters [6], [8]. This enzyme, Pantothenate kinase (CoaA), a homodimer, follows a compulsory ordered mechanism with ATP as the first substrate [9]. The mode of the CoA-effected inhibition was demonstrated by Yun et al. who showed that CoA binds the enzyme at a site similar to that occupied by the phosphate donor, ATP, therefore competitively inhibiting pantothenate kinase [10].

The penultimate enzyme of the biosynthetic pathway, 4′-phosphopantetheine adenylyltransferase, CoaD, has also been implicated in the regulation of the universal CoA biosynthesis. Geerlof et al. demonstrated that purified *E. coli* CoaD co-elutes with 0.5 mole of CoA per mole of the enzyme [11]. Rubio et al. demonstrated that the *Arabidopsis thaliana* CoaD plays a critical role in plant growth, salt/osmotic stress resistance and seed lipid storage [12]. Thus the five-step Coenzyme A biosynthesis pathway has been shown to be regulated at its first step, CoaA and at the penultimate step, CoaD.

The importance of CoA in central metabolism and the vast differences in its biosynthesis in prokaryotic pathogens and eukaryotic hosts have prompted the design and testing of several inhibitors against the individual enzymes of the prokaryotic CoA biosyntheses. A majority of these inhibitors have targeted the two known regulatory steps of the pathway, those catalyzed by CoaA and CoaD. Several structural analogues of the substrate for CoaA, pantothenate, obtained mostly through the modification of the β-alanine moiety of pantothenic acid, for example, replacement of the carboxylic acid group with a sulphonic acid, N-substituted sulphonamide, thiol, disulphide, sulphone, alcohol, ketone, N-substituted amide, N-substituted carbamate or an N-substituted ureido group have shown varied antibacterial effects [13]. Analogues of 4′-phosphopantetheine, the substrate for CoaD, have also shown potent inhibition of the enzyme, however, these did not show effective antibacterial activity as they could not cross the membrane barrier [14]. Therefore, careful design of compounds inhibiting the enzymes of the mycobacterial CoA biosynthesis hold potential for future drug development against tuberculosis.

Not many studies have been undertaken so far to distinguish the role of the last enzyme of the pathway, dephosphocoenzyme A kinase, which ultimately generates the cofactor CoA, in the regulation of metabolite flow through this critical pathway. In view of the unique characteristics of the mycobacterial enzyme in terms of its kinetic properties compared to its bacterial and eukaryotic counterparts, it was interesting to examine the regulation of this catalytic activity. Further, considering the fact that the tubercular pathogen invests a majority of its biosynthetic capability in lipid metabolism for which CoA is a critical cofactor, the pathogen potentially employs novel mechanisms to regulate CoA production in tune with its fastidious requirements for this cofactor and its thioesters [15], [16]. We have previously demonstrated that the last enzyme of mycobacterial CoA biosynthesis is inhibited by the cellular metabolite CTP [17]. Our previous docking analyses further provided an insight into the potential mode of this inhibition, with the enzyme's leading substrate, DCoA and CTP interacting with some common residues on the enzyme and potentially occupying overlapping binding sites on mycobacterial CoaE [17]. Here we elucidate the elegant mechanism employed by this oldest known infectious agent to effect regulation at the last step of the biosynthesis of a critical enzymatic cofactor, entailing a dynamic interplay between the monomeric and trimeric states of the enzyme, effected by the interaction of CoaE with its leading substrate, DCoA and inhibitor, CTP.

## Materials and Methods

### CTP inhibition assays

The kinetic/biochemical activity of the mycobacterial dephosphocoenzyme A kinase was evaluated in the presence of CTP by the spectrophotometric and isothermal titration calorimetric assays described in [17]. For the inhibition assays, the enzyme was pre-saturated with 1 mM CTP for 20 mins before initiating catalysis. For the single-coupled spectrophotometric assay, the reaction mix contained Tris (50 mM), MgCl_2_ (10 mM), á-ketoglutarate dehydrogenase, NAD (0.3 mM), ATP (1 mM), á-ketoglutarate (4 mM) and thiamine pyrophosphate (1 mM).

### CoaE specific activity assays

The specific activity of the mycobacterial CoaE was assessed by the double-coupled pyruvate kinase-lactate dehydrogenase (PK-LDH) assay system described in [17]. The assay mixture contained DCoA (0.5 mM), ATP (1 mM), MgCl_2_ (10 mM), PEP (0.8 mM), NADH (0.3 mM), 50 mM Tris, pH 8.0 and PK-LDH. In order to distinguish the more active species between the trimeric and monomeric forms, the specific activity was measured as a function of enzyme concentration using a range of CoaE concentrations (10 nM–10 µM) at a fixed DCoA concentration (0.5 mM). In order to evaluate the effect of CTP on the specific activity, the enzyme was saturated with 1 mM CTP for 20 mins prior to activity measurements. Enzymatic velocity vs. substrate concentration plots were also plotted using parameters determined by the double-coupled spectrophotometric assay.

### Size exclusion chromatographic analysis

Oligomeric status of the mycobacterial CoaE was determined by size exclusion chromatography (SEC) of 100 µM protein on a Superdex™200 HR10×300 mm column attached to a fast pressure liquid chromatography system (Amersham Pharmacia). The flow rate was maintained at 0.2 mL/min and the absorbance was recorded at 280 nm. SEC was carried out in the presence and absence of 1 mM ATP, DCoA, CoA, CTP, acetyl-CoA, butyryl-CoA, malonyl-CoA, succinyl-CoA, β-hydroxybutyryl-CoA and crotonoyl-CoA. Prior to the mycobacterial CoaE experiments, the column was calibrated with lysozyme (14 kDa), carbonic anhydrase (29 kDa), ovalbumin (45 kDa), bovine serum albumin (66 kDa) and aldolase (158 kDa).

### Analysis by Dynamic Light Scattering (DLS)

DLS studies were performed for the mycobacterial dephosphocoenzyme A kinase on a DynaPro Dynamic Light Scattering instrument. The protein sample (2 mg/mL) in 20 mM Tris, 150 mM NaCl, 10% glycerol (pH 7.8) was centrifuged at 14,000 rpm for 25 min prior to taking measurements. Dynamics V6 software was used to calculate the hydrodynamic radius of mycobacterial CoaE.

### Glutaraldehyde crosslinking of CoaE

To avoid a decrease in the crosslinking yield due to the interaction of the Tris amino groups with glutaraldehyde, CoaE was dialyzed overnight in 1X PBS, pH 7.8. A Ni-NTA agarose resin of 1 mL final volume was specifically packed and equilibrated with Buffer A (10 mM imidazole, 50 mM NaCl, 50 mM phosphate buffer, pH 8.0) for the crosslinking experiments. CoaE (500 µg) was prepared in Buffer A. 4 mL of the sample was then slowly applied to the column. Upon protein binding, the column was washed with 8 mL of Buffer A and 8 mL of Buffer B (50 mM NaCl, 50 mM phosphate buffer, pH 8.0). Glutaraldehyde (2.5 mL) prepared to a final concentration of 0.05% (v/v) in Buffer B was added to the column and allowed to flow through by gravity. The crosslinking reaction was then quenched with 8 mL of 0.5 M Tris/HCl, pH 8.0. Buffer C (8 mL) (300 mM imidazole, 50 mM NaCl, 50 mM phosphate buffer, pH 8.0) was then used to elute the bound crosslinked protein [18]. The covalent oligomers were then resolved on an 8% SDS–PAGE. All steps were carried out at room temperature.

### Cloning, expression and purification of the CoaE cysteine mutants, C235S and C368S

Utilizing the primers, C235S_Fwd: 5′ –GATCGCGTCCGGGCATAAGGCCTTG- 3′ and C235S_Rev: 5′ –ATGCCCGGACGCGATCTTTAG- 3′ and the set, C368S_Fwd: 5′ –GAAGACTATTTGACGGTCAAGTCTGACGCCGACAG- 3′ and C368S_Rev: 5′-GTCGGCGCGCCTGTCGGCGTCAGACTTGACC- 3′, the CoaE cysteine mutants, C235S and C368S, were PCR-amplified from the mycobacterial genomic DNA using the same conditions as the wild type protein described in [17]. The cysteine mutant proteins were expressed and purified like wild type CoaE [17], [19].

### Effects of reducing agents on CoaE oligomerization

The effect of a range of DTT concentrations (20 µM–20 mM) and two β-ME concentrations (2 µM and 10 mM) was scanned on purified mycobacterial CoaE, its active site mutants (D33E, L113A, K14A) and cysteine mutants (C235S, C368S) at 20ìg concentrations in 50 µL reactions [15]. Each enzyme was incubated with the reducing agents over a range of time periods (10′, 20′, 30′, 45′ and 60 mins) in order to assess if prolonged incubation has any effect on the oligomeric status. In two different sets of reactions, CoaE was pre-incubated with 1 mM DCoA and 1 mM CTP for 30 mins before incubation with the reducing agents to determine the protection afforded by these metabolites on the enzyme towards disulphide-reduction. Upon reduction, a range of H_2_O_2_ concentrations (50 µM–20 mM), incubated with the reduced enzyme for 30 mins, were also scanned to study reduction-reversal. The oligomeric status of the reaction products was then evaluated by the addition of non-reducing Laemmli SDS-PAGE buffer, sample boiling for 5 min and separation on an 8% non-reducing SDS-PAGE and a 8% Native PAGE. Both gels were Coomassie-stained. Further confirmation of the results was obtained by Western blotting of the gels with anti-His antibodies.

### Limited proteolysis of mycobacterial dephosphocoenzyme A kinase

The mycobacterial enzyme was subjected to proteolytic cleavage with three different proteases, trypsin, chymotrypsin and subtilisin. 4 ng/µL of trypsin was used to cleave 10 µM of the enzyme at 25°C. Prior to incubation, the master mix was subdivided into individual aliquots, one of which was withdrawn at fixed time intervals. The proteolytic reaction was monitored for 180 minutes and one sample was incubated overnight at 25°C. These samples were resolved on an 8% SDS gel which was Coomassie-stained to assess the fragmentation pattern. To study the protection afforded by the ligands on the enzyme, ATP, DCoA, CoA and CTP were incubated with the mycobacterial CoaE for 30 mins on ice, prior to the addition of trypsin.

### Fluorescence emission analysis of CoaE

Fluorescence measurements were performed on a JobinYvon Horiba fluorimeter, exciting the proteins at 295 nm and recording the fluorescence emission from 300–450 nm. The excitation and emission monochromator slit widths were 2.5 nm and 5 nm, respectively. The proteins were dialyzed in 1X PBS and 5% glycerol and were used at concentrations of 5–10 µM. Measurements were done in a 100 µL quartz cuvette. Spectra were corrected for background fluorescence (almost negligible) from the buffer, DCoA and CTP in all experiments.

## Results

### The role of CTP in the regulation of CoaE activity

**Figure 1:**
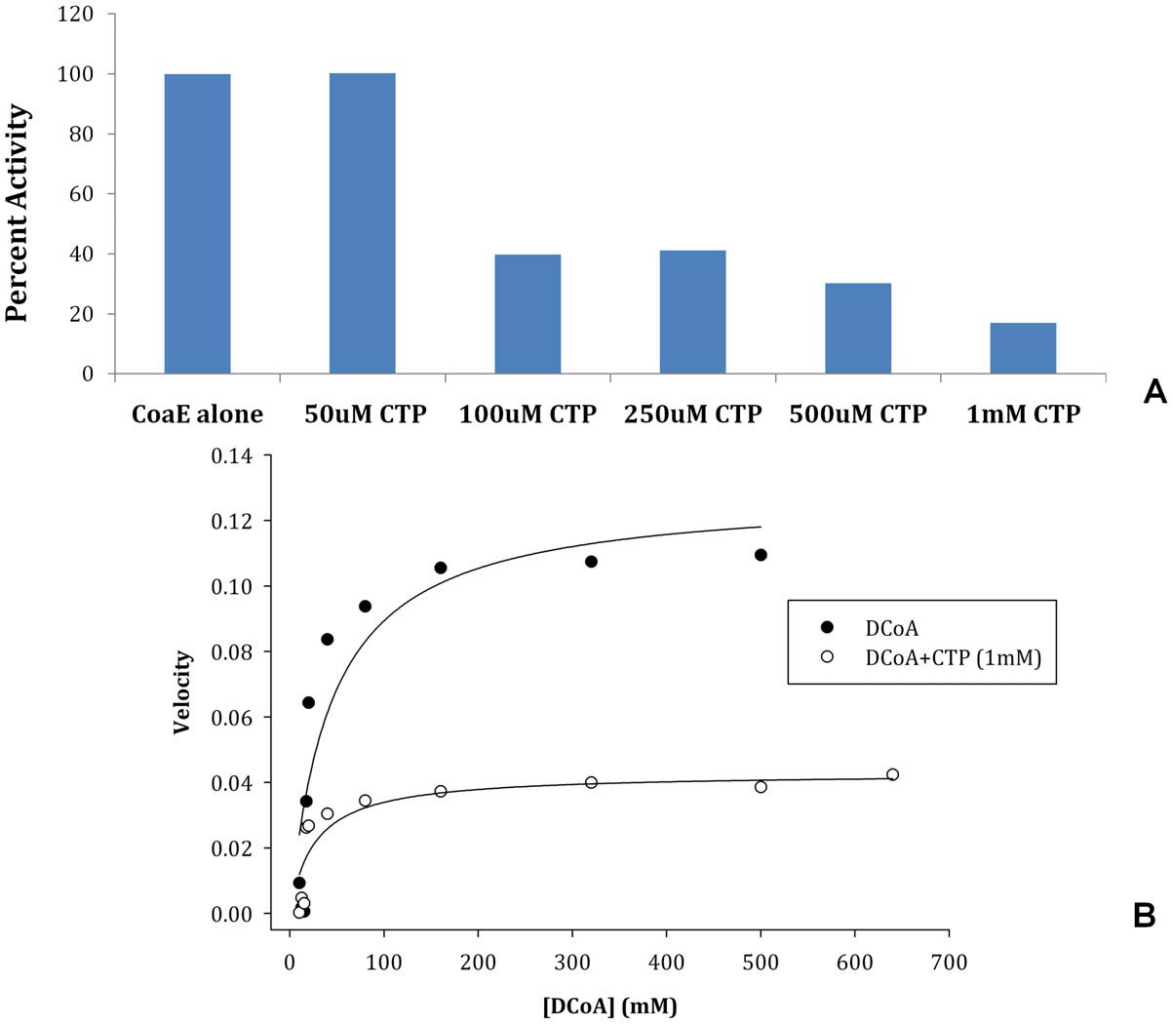
Effects of CTP on CoaE enzymatic activity. (A), Histogram depicting the percent decrease in CoaE activity in the presence of CTP, with the activity of CoaE in the presence of ATP (1 mM) and DCoA (0.5 mM) treated as 100% (Lane 1). Lanes 2–6 show the percent activity of CoaE in the presence of different concentrations of CTP; Lane 2, 50 µM CTP; Lane 3, 100 µM CTP; Lane 4, 250 µM CTP; Lane 5, 500 µM CTP; Lane 6, 1 mM CTP. (B), CoaE reaction velocity plotted versus leading substrate, DCoA concentrations (10–640 µM) in the presence and absence of CTP (1 mM), measured using the coupled PK-LDH spectrophotometric assay.

As previously demonstrated, of all the phosphate donors tested for the mycobacterial CoaE, CTP bound the enzyme the most tightly [17]. However, this nucleotide did not participate in catalysis. Following up on the lead of strong binding of CTP to the enzyme, its effect on CoaE activity and catalysis was further examined. As shown in Figure 1A, pre-incubation of the mycobacterial CoaE with different concentrations of CTP resulted in an inhibition of the enzymatic reaction, pre-incubation with 1 mM CTP causing a decline in activity to about 16% of that seen in its absence. A Michealis-Menten V vs. [S] plot of the velocity of the CoaE enzymatic reaction vs. a range of DCoA concentrations in the presence and absence of CTP (1 mM) is shown in Figure 1B.

### The role of the oligomeric status of CoaE in its regulation

The mycobacterial dephosphocoenzyme A kinase belongs to the P-loop containing nucleoside monophosphate kinase family (NMPK) of the nucleoside triphosphate hydrolases superfamily, majority members of which are structurally very similar despite sharing very low sequence homology. The crystal structures solved for the three bacterial CoaEs till date give evidence for a monomeric state for the enzymes with the *E. coli* enzyme (being a monomer in solution) having crystallized as a crystallographic trimer due to the presence of sulphate in the crystallization buffer [20]. Considering the unique properties of the mycobacterial CoaE and its differences from the other dephosphocoenzyme A kinases, its oligomeric status was studied.

### Analysis by Size-Exclusion Chromatography

#### The oligomeric equilibrium of the native enzyme

**Figure 2:**
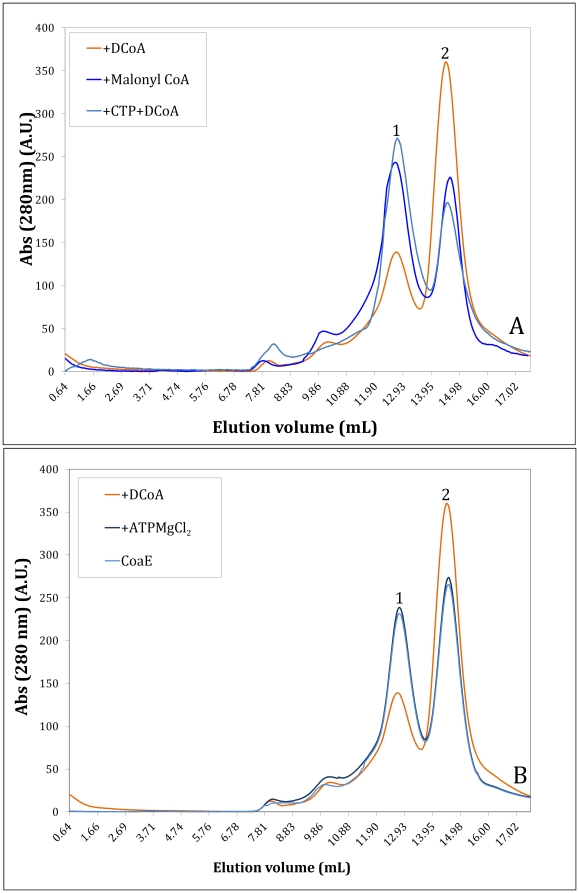
Determination of the oligomeric status of CoaE by size exclusion chromatography. (A), Each individual reaction mixture, incubated for 30 mins, contained CoaE alone; CoaE+1 mM DCoA; CoaE+1 mM ATP+1 mM MgCl_2_ was loaded on a Superdex S-200 column. (B), Each individual reaction mixture, incubated for 30 mins, contained CoaE+1 mM DCoA; CoaE+1 mM CTP (incubated for 30 mins) + DCoA (incubated for another 30 mins); CoaE+ 1 mM Malonyl-CoA. Peak 1: Trimeric CoaE (M_r_ ∼140,000±2900 Da), Peak 2: Monomeric CoaE (M_r_ ∼47,000±15 Da) (Molecular masses expressed as mean ± S.D. of five independent experiments).

On a Sephadex-200 column, the native protein eluted as a mixture of 2 peaks of approximately similar abundance, which were assigned as a trimer (Mr ∼141,000±150 Da, peak 1) and a monomer (Mr of ∼47,000±150 Da, peak 2) (on the basis of the position of the elution peaks of proteins used as standards to calibrate the column) with a trimer: monomer ratio of 1∶(0.932) (Figure 2A). Since such an equilibrium between two oligomeric states has not been reported for any other dephosphocoenzyme A kinase, the role of this unique feature of the mycobacterial enzyme was explored further.

#### The effect of the leading substrate, dephosphocoenzyme A

The effects of DCoA on the oligomerization of the enzyme were explored through gel filtration. Interestingly, when the native protein was incubated with DCoA for 30 mins at 4°C and then run on the column, a consistent marked increase in the abundance of the monomeric peak with a consequent decrease in the trimeric peak intensity was observed; the trimer: monomer ratio now being 1∶(2.14) (Figure 2A). This significant shift from the trimeric to the monomeric state was again observed when the eluted trimeric peak was collected, incubated with DCoA and re-injected (as seen in five independent experiments). In order to evaluate the effect of the second substrate, ATP, on the re-adjustment of the oligomeric equilibrium of the mycobacterial CoaE, the enzyme was incubated with the phosphate donor for 30 mins before gel filtration. Surprisingly, pre-incubation with ATP did not have any effect on the distribution of the enzyme between the two oligomeric states (Figure 2A). The failure of ATP to effect any change in the oligomeric status of the enzyme can be explained in the light of the fact that ATP binds the enzyme well only when the enzyme is *a priori* saturated with DCoA [17].

In order to rule out the above-mentioned re-equilibration of the oligomeric states as a non-specific consequence of the interaction of the enzyme with an analog of CoA or its thioesters, the enzyme was separately incubated with 1 mM each of acetyl-CoA, malonyl-CoA, crotonoyl-CoA, succinyl-CoA and -hydroxybutyryl-CoA and run on the S-200 column. None of the CoA thioesters elicited a response remotely similar to that observed with DCoA, as CoA or its thioesters did not alter the oligomerization status of CoaE. A representative curve with malonyl-CoA is shown in Figure 2B where the trimer: monomer ratio is 1∶(1.01), similar to that of the native enzyme. Therefore the aforementioned effect of DCoA on the enzyme's oligomeric status is not a non-specific consequence effected by any CoA species but is an effect of the interaction of the leading substrate with the enzyme.

#### The effect of the inhibitor, CTP, on the CoaE oligomeric status

The mechanism of CTP inhibition of the mycobacterial CoaE activity was further probed by exploring whether this inhibition in any way translated into a shift in the oligomeric status. Pre-incubating CoaE with 1 mM CTP alone showed a trimer: monomer ratio of 1∶(0.614) as against a ratio of 1∶(0.932) observed for the native CoaE (Figure 2B). Significantly, pre-incubation of the enzyme with 1 mM CTP, prior to its incubation with DCoA, prevents the DCoA-mediated CoaE transition from a trimeric state to the monomer, with 38% of the enzyme eluting as a monomer in the presence of both CTP and DCoA as against 70% of the monomer in the presence of DCoA (Figure 2B). Thus, the transition of the enzyme from its potentially inactive, trimeric state to its active, monomeric form, originally effected by the leading substrate, DCoA, is prevented by pre-incubation with CTP.

### Analysis by Dynamic Light Scattering

A further confirmation of the enzymatic transitions of CoaE in the presence of the ligand effectors was obtained by carrying out DLS measurements on the enzyme which revealed three distinct populations visualized as three peaks in the histogram, corresponding to the monomer (2.8 nm radius), the trimer (5.7 nm radius) and a non-specific aggregate (a radius of 30 nm and above) respectively with the aggregate being the most thinly populated, forming a mere 5–7% of the protein population distributed in the three states. The aggregate was not observed in many DLS experiments. An increase in the intensity of the monodisperse peak corresponding to the monomer with a hydrodynamic radius of 2.8 nm when the enzyme was pre-incubated with DCoA was observed. Moreover, these DLS data also corroborate the influence of CTP on the oligomerization status of CoaE as elucidated above by SEC, demonstrating that CoaE pre-incubation with CTP shows a similar distribution of the peaks as those of the native enzyme alone.

### Glutaraldehyde crosslinking of the mycobacterial CoaE

**Figure 3:**
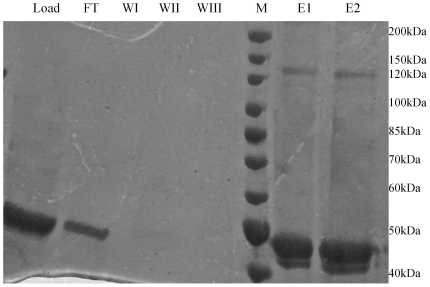
The on-column glutaraldehyde crosslinking of the mycobacterial CoaE. The 8% SDS-PAGE gel picture shows the CoaE protein loaded on the column (Load); the flowthrough from the column during loading (FT), the eluate during the wash steps (WI, WII and WIII); the molecular weight marker (M) and the elution aliquots (E1 and E2).

Glutaraldehyde crosslinking experiments on CoaE further confirmed the presence of the enzymatic trimer through the appearance of a band of ∼140 kDa corresponding to the trimeric molecular mass of the mycobacterial CoaE (Figure 3).

### Effects of reducing agents on the oligomeric status

The mycobacterial CoaE has three cysteine residues and the final confirmation of its oligomeric re-equilibration was obtained by ruling out a cysteine-mediated covalent oligomerization of CoaE. The lack of reduction-sensitivity of the oligomerization and the absence of any effect of Cys-Ser mutagenesis (C235S, C368S) confirmed that CoaE oligomerization was not a disulphide-mediated covalent process.

### CoaE specific activity assays

**Figure 4:**
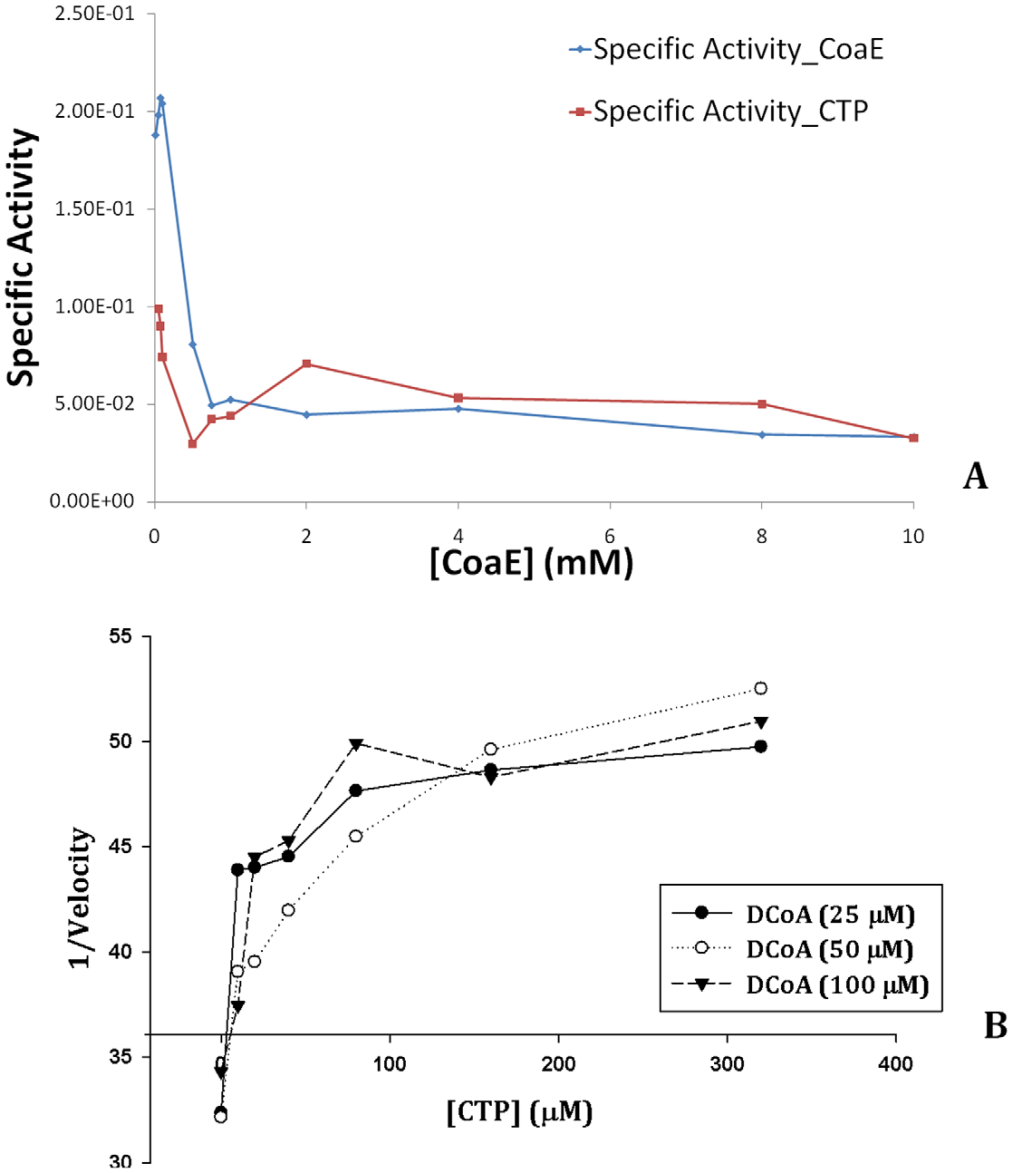
Inhibition kinetics. (A), Measurement of the specific activity of CoaE as a function of enzyme concentration. Freshly prepared CoaE was diluted in buffer alone or was pre-saturated with 1 mM CTP and diluted in buffer+CTP to a range of concentrations (10 nM–10 µM) and activity was measured at 0.5 mM DCoA. (B), Dixon plot (1/V vs. [CTP]) illustrating the inhibition of the mycobacterial dephosphocoenzyme A kinase by CTP (Ki = 34 µM), at three different concentrations of the leading substrate, DCoA.

The equilibrium distribution of an enzyme in various oligomeric states is affected by the enzymatic concentration. Therefore, dilution of the enzyme is bound to have an effect on its specific activity as the change in enzyme concentration will reflect in the distribution of the enzyme in the active and inactive species. Thus, in order to conclusively identify the active form of the mycobacterial CoaE among the two oligomeric states and gain an insight into the form that is potentially active physiologically, the specific activity of the enzyme was studied as a function of CoaE concentration (10 nM–10 µM), keeping the concentration of the leading substrate DCoA constant (0.5 mM). It is interesting to note that CoaE showed a higher specific activity at low enzyme concentrations i.e. as the enzyme concentration decreases, the addition of a fixed concentration of DCoA brings about a consistent increase in the specific activity, demonstrating that the monomer is the active species (Figure 4A). Thus, the monomeric form of the mycobacterial CoaE is, in all probability, the active form of the enzyme in the cell. Further, the effect of CTP on catalysis was assessed by incubating the enzyme with 1 mM CTP which resulted in a decrease in the values of the specific activity measured at lower CoaE concentrations, this effect being apparent when the enzyme was most dilute (Figure 4A). It should be noted that the reaction mixture for the assay was compensated appropriately each time the enzyme was diluted in the presence of CTP so as to maintain the final CTP concentration constant at 1 mM. This assay is only limited by the range of enzyme concentrations tested viz., the inability to record catalysis at low concentrations due to a reduced sensitivity of product detection and the complexity in maintaining initial rate conditions at high enzyme concentrations. Further, a Dixon plot (1/V vs. [I = CTP] at different [S = DCoA] assigned a Ki value of 34 µM to this unique metabolic inhibitor, CTP (Figure 4B) which shows competitive inhibition of mycobacterial CoaE. It is interesting to note that the Ki is quite similar to the affinity of the enzyme (Km) for its leading substrate, DCoA [17].

#### Ligand-induced conformational changes in CoaE

In order to ascertain how the effects of the ligands, DCoA and CTP, on the catalytic activity and oligomeric equilibrium actually translate into changes in the gross secondary and tertiary structure of the enzyme, two different approaches were employed.

#### Limited Proteolysis of the mycobacterial CoaE

**Figure 5:**
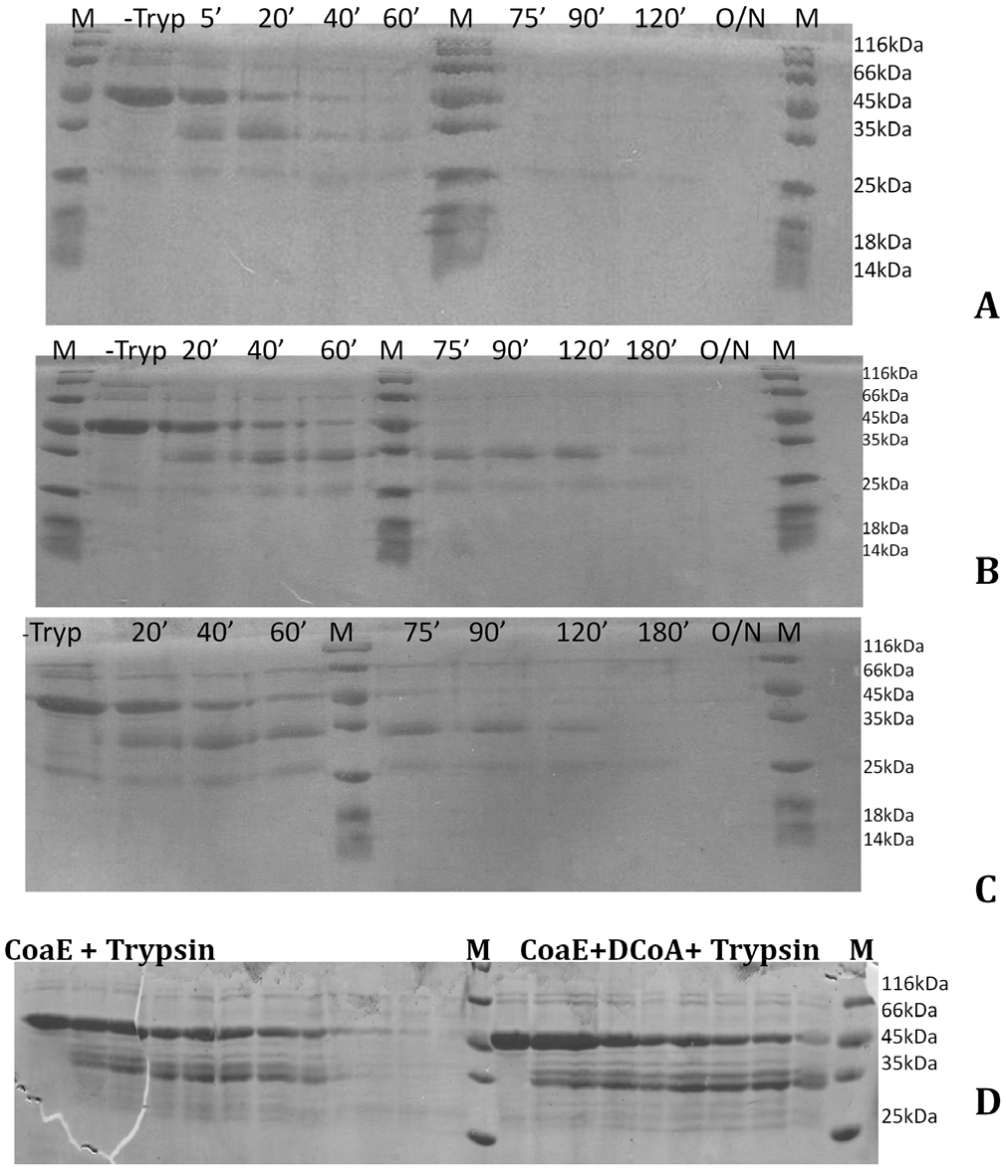
Limited proteolysis of the mycobacterial CoaE studied by tryptic cleavage at various time points and separation of the digestion products on an 8% SDS-PAGE gel. (A), tryptic digestion fragmentation pattern for CoaE. (B), protection afforded by the leading substrate, DCoA, on the mycobacterial enzyme during proteolysis. (C), fragmentation pattern of CoaE digestion in the presence of CTP. (D), Comparison of the cleavage patterns of CoaE alone and that in the presence of DCoA. (-Tryp) denotes the enzyme aliquot in the absence of the protease.

Limited proteolysis helps study the change in the accessibility of scissile peptide bonds, thereby shedding light on the mechanisms of enzymatic catalysis and regulation. Thus, to study the effects of the leading substrate, DCoA and the inhibitor, CTP on the enzyme in terms of gross conformational changes effected, the protection afforded by these ligands upon the enzyme during limited proteolysis studies was analyzed. Mycobacterial CoaE was proteolyzed by three different proteases, trypsin, subtilisin, chymotrypsin, under limiting conditions at a protease to protein ratio of 1∶50 (w/w). Although CoaE has 46 theoretical tryptic cleavage sites, upon digestion with trypsin only a limited number of fragments were generated (Figure 5A and D).

#### Protection afforded by DCoA during proteolysis

Proteolysis of 10 µM CoaE in the presence of 300 µM DCoA demonstrated that DCoA stabilizes an ∼34 kDa proteolyzed fragment (Figure 5B) as late as 120 mins post-incubation with the protease. By virtue of DCoA binding to the N-terminal domain of the bi-domain mycobacterial CoaE, this ∼34 kDa fragment is in all possibility the NTD of the enzyme. It is interesting to note that this particular fragment does not persist in the tryptic digest of the native enzyme alone, with the band for this fragment disappearing totally between 60–75 mins upon incubation with trypsin. Thus, DCoA binding to mycobacterial CoaE possibly induces conformational changes in the enzyme which obscure the scissile bond previously available for tryptic cleavage protecting the enzyme from proteolytic digestion.

#### Protection afforded by CTP during proteolysis

It is interesting to note that limited proteolysis of the mycobacterial CoaE (10 µM) in the presence of the regulator, CTP (1 mM), yields a similar fragmentation pattern as observed in the presence of DCoA (Figure 5C). CTP also protects the same fragment from further proteolysis as was seen with DCoA, with the band persisting up to 120 mins in the presence of CTP as against a mere 60–75 mins in the native enzyme digestion. Thus, CTP affords similar protection on the mycobacterial enzyme as the leading substrate, hinting at a similar site of binding of the two substrates.

#### Fluorescence studies on CoaE

**Figure 6:**
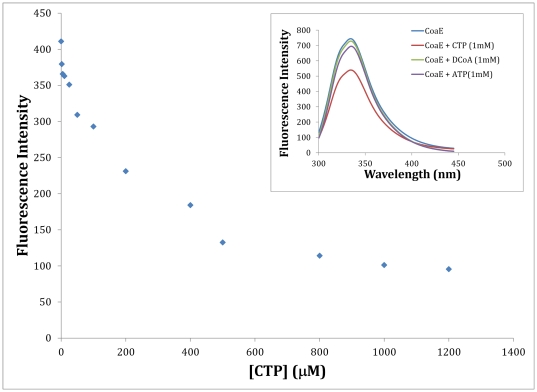
Fluorescence studies. The effect of CTP on CoaE fluorescence depicted by a plot of the fluorescence emission intensity recorded at the maxima of 337.5 nm versus the various concentrations of CTP used. (Inset), The effects of the ligands, DCoA, ATP and CTP on the fluorescence emission of mycobacterial CoaE.

The mycobacterial CoaE possesses 11 tryptophan residues which can therefore be employed to study the overall conformational changes induced upon DCoA and CTP binding. In the absence of any ligand, the emission spectrum is a broad peak with an emission maximum centered at 337.5 nm when it is excited at 295 nm (Figure 6 inset). The Lmax of a comparable concentration of L-Trp solution was 355 nm. The blue-shifted Lmax in CoaE indicated that the tryptophan residues are on average in a nonpolar (hydrophobic) environment. Though increasing concentrations of DCoA show no effect on the tryptophan emission spectrum of 10 µM CoaE at pH 7.8 (Figure 6 inset), saturating concentrations of CTP cause a quenching of fluorescence indicating a decreased exposure to the solvent of the tryptophan residues in the enzyme-ligand (CoaE-CTP) complex (Figure 6). Thus, CTP binding induces conformational changes which affect the tryptophan environment in the mycobacterial CoaE.

## Discussion

Vitamin and cofactor biosynthetic pathways hold considerable promise for anti-mycobacterial therapy. Pioneering work from the Jacobs' lab, for example, has shown that a *Mycobacterium tuberculosis* pantothenate auxotroph (ΔpanC-ΔpanB) has impaired survival and pathogenesis, the highly attenuated knock-out strain imparting long-term protection to mice against tuberculosis [15], [16]. The CoA biosynthetic pathway, unlike that of its precursor pantothenate, is a ubiquitous and essential pathway in a vast majority of organisms. Synthesizing an essential metabolite, the flux through this pathway is therefore very tightly regulated with each of the five steps leading to CoA biosynthesis being regulated by multiple mechanisms to ensure efficient utilization of cellular resources. The last enzyme of the pathway, dephosphocoenzyme A kinase, has surprisingly received very little attention over the past several years and there are no reports on the regulation of this enzymatic activity so far. Considering the fact that it is the last step that eventually gives rise to CoA and that this enzyme from the mycobacterial system varies strikingly from its human counterpart, the significance of regulation of this enzyme needs no emphasis. In this study, the molecular determinants for the oligomeric assembly of CoaE were dissected and the role of the oligomerization status in substrate binding, catalysis and kinetic regulation was analyzed.

CoaE belongs to the family of nucleotide and nucleoside kinases, majority members of which are monomeric. As seen in these studies, the native mycobacterial enzyme exists primarily as a mixture of a trimer and a monomer of almost equal abundance but incubation with the leading substrate, DCoA, causes a shift of the trimer towards the monomeric state. The second substrate, ATP, alone has no effect on the enzyme's oligomeric status and this is further validated by our previous ITC studies, which demonstrate ATP's inability to bind the enzyme in the absence of DCoA [17]. Interestingly, the aforementioned effect of DCoA is not a non-specific consequence effected by any CoA species as neither CoA nor its thioesters are capable of mediating such a change and therefore it is an unambiguous effect of the interaction of the leading substrate with the enzyme. Further, kinetic assays unequivocally established that the monomeric form is the more active form of the enzyme. It can therefore be hypothesized that in the cell, the enzyme is possibly sequestered in an inactive higher trimeric form which gets converted to the active, monomeric form in the presence of the leading substrate, DCoA, which then facilitates the binding of the second substrate, ATP. This hints at a possible regulatory mechanism the cell employs to check production of the essential metabolite CoA by keeping the ultimate enzyme of its biosynthetic pathway catalytically dormant till the need arises.

The role of the dynamic interplay of the various oligomeric states of an enzyme in its regulation is not unusual. The mycobacterial CoaE shows behavior analogous to the key enzyme of the mammalian sialic acid biosynthesis, UDP-N-acetylglucosamine 2-epimerase/N-acetylmannosamine kinase (GNE). The activity of GNE is regulated by a series of mechanisms; (i) allosteric feedback inhibition by CMP-Neu5Ac, (ii) competitive inhibition by UDP and (iii) re-equilibration of various oligomeric states (monomers, dimers, tetramers and higher aggregates). The epimerase substrate, UDP-GlcNAc favors the formation of the tetramers which is the active form in the cell, while in the presence of the allosteric inhibitor CMP-Neu5Ac, the enzyme is in the dimeric state. The presence of both the ligands favors a tetrameric GNE [21]. These results are similar to those observed for the mycobacterial CoaE. Interestingly, despite it being counter-intuitive that monomers and not the readily-formed oligomers (which can possibly allow structural and functional variations) form the optimally active species, there are several examples of enzymes which are active in their monomeric forms, such as the rat liver UMP/CMP kinase with which the mycobacterial CoaE shares significant homology, the purine nucleotide phosphorylase from bovine spleen, the *E. coli* ribonucleoside diphosphate reductase etc. [22]. Therefore, even though the involvement of different oligomeric states in activity regulation has not been investigated for the Coenzyme A biosynthesis, the suggestion that the monomeric form of the enzyme is the most active is not uncommon in biology.

**Figure 7:**
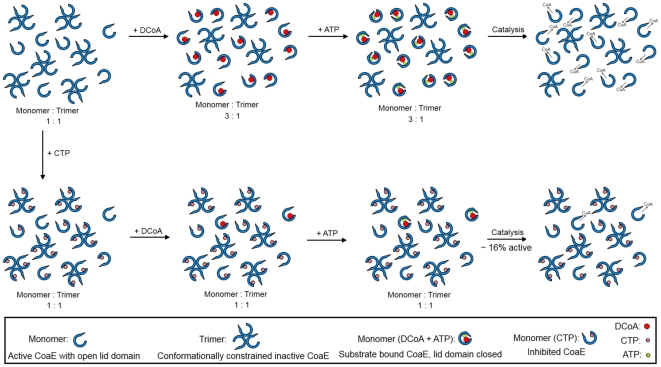
Schematic depicting the dynamic interplay between the enzyme's leading substrate, DCoA and the cellular metabolite, CTP, in regulating the activity of the last enzyme of mycobacterial CoA biosynthesis. The enzyme is sequestered in a trimeric state that renders it inactive, potentially due to spatial constraints imposed on the mobile lid and CoA domains. DCoA, upon binding, induces monomerization, releasing these constraints and inducing conformational changes in the enzyme, allowing the phosphate donor, ATP, to bind and catalysis to occur. CTP, on the other hand, prevents DCoA from binding the enzyme by virtue of occupying a similar binding site on the enzyme, thereby preventing the oligomeric re-equilibration, reflected in a mere 16% active CoaE in the presence of 1 mM CTP. Thus the mycobacterial cell employs regulation at the last enzyme of CoA biosynthesis via two co-acting mechanisms.

The oligomeric re-equilibration observed for the mycobacterial CoaE could perhaps be a universal regulatory mechanism exploited by all dephosphocoenzyme A kinases in light of the fact that the *E. coli* enzyme was crystallized as a trimer while in solution a majority was found to exist as a monomer as were the *Haemophilus* and *Thermus* CoaEs [20], [23]–[24]. The *E. coli* crystal structure probably captured the potentially dormant higher oligomer in the crystal which was converted into the active monomer in the presence of the substrate during activity analyses by HPLC, as the authors clearly point out that ‘gel filtration and DLS measurements on the purified protein before crystallization indicated a monomeric state in solution’ [20]. This is further corroborated by the fact that an analysis of the *E. coli* CoaE biochemical activity in solution by HPLC in an earlier study showed it to be a monomer [25]. It can therefore be hypothesized that the P-loop-containing DCoA kinases which are known to possess mobile lid and CoA domains, are maintained in the inactive state as higher oligomers (i.e. as trimers) which limit the mobility of the lid and CoA domains due to spatial constraints and therefore render these oligomers incapable of catalysis by preventing the phosphate transfer to the ever-present water (Figure 7). These inactive oligomers are then recruited by the cell when CoA is required. DCoA, being the leading substrate, paves the way for the beginning of catalysis by releasing the spatial constraints, thereby transforming the inactive trimers to active monomers for catalysis (Figure 7) [17].

The strong binding of CTP to the mycobacterial CoaE despite its inability to serve as a phosphate donor in the kinase reaction *a priori* appears puzzling [17]. The role of this unique cellular metabolite in the regulation of CoaE activity was therefore investigated in detail. Similar regulation of metabolic enzymes by NTPs other than their natural substrates is not uncommon, one such example being reported in the pyrimidine nucleotide biosynthesis pathway and like CoA biosynthesis is essential for the survival of several organisms. In the *E. coli* pyrimidine nucleotide biosynthesis, the hexameric uridyl monophosphate kinase has been shown to be activated by GTP and inhibited by UTP [26]. Further, it is interesting to note that the mycobacterial dephosphocoenzyme A kinase, like the bacterial uridyl monophosphate kinase, also belongs to the NMPK superfamily. Therefore, the relevance of the strong interaction of CTP with the mycobacterial dephosphocoenzyme A kinase was explored further. It is interesting to note that pre-incubating the mycobacterial CoaE with CTP inhibited the kinase. These results were clearly explained by the SEC data which show that CTP very neatly sequesters the enzyme in the trimeric state, thereby preventing DCoA from initiating monomerization. Therefore, the percentage of active enzyme falls, as does the enzyme activity. This is further corroborated by the effects of CTP seen on the measurement of CoaE specific activity as a function of the enzyme concentration. Docking both the metabolic inhibitor, CTP and the leading substrate, DCoA, clearly reveals that there is an overlap in the two binding sites on the mycobacterial CoaE thereby explaining the inhibition shown by CTP of the activation by DCoA and therefore the conversion of the inactive trimer to the active monomer [17]. Therefore, the data presented here hint at a potential regulatory mechanism whereby CTP, upon binding the last enzyme of the CoA biosynthetic pathway, prevents the latter's interaction with its leading substrate, thereby preventing its oligomeric transformation to the active form; in effect, limiting the catalytic efficiency of CoaE (Figure 7). While the data do not allow us to conclude generality of this regulation, the data presented here suggest that the tubercular pathogen seems to have evolved a neat regulatory mechanism at a critical step of central metabolism which elegantly modulates metabolic flux through this step of the pathway by subtle changes in the percentage of active enzyme available, mediated through a barrier lifted only by the presence of the leading substrate. Thus, the dynamic interplay between the two oligomeric states of the mycobacterial CoaE regulates enzyme activity. The organism has further evolved a second level of regulation via the cellular metabolite CTP which keeps the oligomeric equilibrium in check. It is tempting to suggest that analogues of CTP would prove to be promising as inhibitors of *Mycobacterium tuberculosis* growth.

The effects of the two ligands, the leading substrate, DCoA and the modulator, CTP, on the conformation of the mycobacterial CoaE have been verified through a variety of approaches. The oligomeric status has been confirmed by DLS measurements and glutaraldehyde crosslinking experiments both of which show evidence of a trimeric state. Conformational changes induced upon the binding of the two ligands have been mapped by limited proteolysis and fluorescence spectroscopic analysis. However, this hypothesis needs further verification through detailed structural analysis of the mycobacterial CoaE.

It is important to note that mycobacterial cellular CoA pools vary widely in response to various stresses [6], [27]–[28]. For example, under the stress conditions faced by mycobacteria in the host, such as a regular onslaught of drugs, whereby the organism shifts to the latently active persistent state with only a handful metabolic activities functioning, delicately fine-tuning its CoA stores is essential for precious energy conservation. Therefore, apart from the regulation effected via oligomeric re-equilibration between the inactive and active enzyme forms, the second layer in the regulatory cascade of CoA generation effected through the influence of CTP on the oligomeric status of CoaE and thereby its activity, effectively limiting CoA biosynthesis, has potentially evolved in mycobacteria to ensure a strict regulation of their response to stress. Thus these data emphasize that, considering its role in fine-tuning the levels of the cellular CoA pool by virtue of two different regulatory processes, the last step of CoA biosynthesis merits greater attention in the overall regulation of the pathway than has been previously allocated.
